# Analysis of antiplatelet therapy adherence in patients with ischemic cerebral stroke

**DOI:** 10.1002/brb3.2982

**Published:** 2023-04-16

**Authors:** Jie Zhong, Yuguang Gao, Deqing Huang, Yueqiang Hu, Qianchao He, Limei Diao, Yuying Hu, Wei Chen

**Affiliations:** ^1^ Department of Neurology, The First Affiliated Hospital Guangxi University of Chinese Medicine Nanning China; ^2^ Department of Emergency, The First Affiliated Hospital Guangxi University of Chinese Medicine Nanning China

**Keywords:** adherence, aspirin, ischemic cerebral stroke, medical insurance, risk model

## Abstract

**Background:**

The related factors affecting the adherence of ischemic cerebral stroke (ICS) patients to antiplatelet therapy have attracted much attention.

**Methods:**

Patients with ICS (confirmed by CT or MRI) were enrolled from January 2020 to July 2021. The demographic data were retrospectively investigated and analyzed. The adherence calculation was as follows: Adherence = number of tablets taken/number of tablets needed to be taken. Adherence < 100% was defined as nonadherence. Severe nonadherence is defined as adherence ≤ 75%.

**Results:**

A total of 229 patients with ICS were enrolled. We found no significant difference in the proportion of patients with nonadherence, while the proportion of severe nonadherence in the aspirin group was significantly higher (*p* < .001). Multivariable analysis indicated that medical insurance (odds ratio [OR] = 0.071, *p* < .001) and regular exercise (OR = 0.438, *p* = .015) were independent factors associated with adherence. In addition, only medical insurance (OR = 5.475, *p* < .001) and aspirin treatment (OR = 0.228, *p* < .001) were independent risk factors associated with severe nonadherence. We therefore constructed a nomogram plot and a model as follows: Adherence risk score = 3 × medical insurance + regular exercise. Patients were divided into low‐risk and high‐risk groups for adherence based on the median model score. A total of 13.3% of patients in the low‐risk group were nonadherent patients compared with 53.4% in the high‐risk group (*p* < .001). Similarly, 8.4% of patients in the low‐risk group had severe nonadherence compared with 19.9% in the high‐risk group (*p* = .022). Moreover, in low‐risk patients, no significant difference was observed. In patients with high risk, aspirin‐treated patients showed significantly decreased adherence compared with the other two groups.

**Conclusion:**

Medical insurance and regular exercise were independent factors for antiplatelet therapy adherence. For patients with high model scores, timely intervention is necessary.

## Graphical abstract

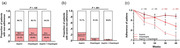

## 1 INTRODUCTION

Ischemic cerebral stroke (ICS) is the most common type of cerebrovascular disease (Feske, 2021; Herpich & Rincon, 2020; Phipps & Cronin, 2020) and accounts for nearly 70% of strokes in China (Wang et al., 2017). The high morbidity and high disability rate of ICS bring heavy economic and social burdens to patients (Ding et al., 2022; Jiang et al., 2020). A previous study indicated that in 2013, stroke was the second leading cause of death (11.8%) worldwide after acute myocardial infarction (14.8%) (Feigin et al., 2017). ICS is also the third most common cause of disability (4.5%) after ischemic heart disease (6.1%) and oncological disorders (Feigin et al., 2017; Florescu et al., 2019). After an acute phase of ICS, long‐term antiplatelet therapy is essential for the secondary prevention of stroke recurrence and related complications.

Aspirin and clopidogrel are commonly used antiplatelet drugs. However, the pros and cons of the two are still controversial. The CAPRIE study showed that 5.32% of patients treated with clopidogrel experienced a risk of primary outcome vascular events (such as ischemic stroke or myocardial infarction and even death) compared with 5.83% of patients treated with aspirin (CAPRIE Steering Committee, 1996). However, when evaluating the benefits of antiplatelet therapy in patients, in addition to analyzing its efficacy and safety, patient adherence also needs to be considered. In particular, it has been confirmed that patients with poor adherence have a worse prognosis (Sakr et al., 2022). However, in practice, the adherence of patients with ICS on antiplatelet therapy is poor, and drugs are often missed during the treatment process (Sakr et al., 2022). The related factors affecting the adherence of ICS patients to antiplatelet therapy have attracted much attention.

Hence, we conducted a retrospective study. We enrolled ICS patients with antiplatelet therapy after the acute phase of disease. Our aim was to explore the differences in adherence among different antiplatelet treatments, as well as to find the relevant factors affecting adherence to antiplatelet therapy. Our results can provide evidence for accurately identifying ICS patients with poor adherence, improving their adherence to antiplatelet therapy, therefore reducing the incidence of secondary stroke and thus improving the prognosis.

## 2 PATIENTS AND METHODS

### 2.1 Patients studied

This study enrolled patients with ICS (confirmed by CT or MRI) who were diagnosed and treated in the First Affiliated Hospital of Guangxi University of Traditional Chinese Medicine from January 2020 to July 2021. All patients were prescribed antiplatelet drugs. Patients were excluded if they were (1) pregnant or lactating women; (2) had a history of gastrointestinal bleeding or recent major surgery; (3) were allergic to aspirin or substances containing salicylic acid or clopidogrel; (4) had hemophilia or thrombocytopenia; (5) had severe organ function abnormalities; (6) had mental illnesses; or (7) had missing key data. The ethics committee of the First Affiliated Hospital of Guangxi University of Traditional Chinese Medicine approved this study, and all enrolled patients provided informed consent.

### 2.2 Laboratory testing

The demographic data of ICS patients were retrospectively investigated and analyzed. Antiplatelet regimen, alcohol consumption history, and tobacco smoking history were also studied. We extracted the records of drug adherence from all patients at each 12‐week follow‐up. The number of tablets taken was acquired by patient self‐report and confirmed with tablet kit at each visit. The adherence calculation is as follows: Adherence = number of tablets taken/number of tablets needed to be taken. Adherence < 100% was defined as nonadherence. Severe nonadherence is defined as adherence ≤ 75%.

### 2.3 Statistical analysis

The measurement units were expressed as the mean ± SD for normally distributed data. Categorical data were expressed as a percentage. One‐way analysis of variance, chi‐square, and Student's *t*‐test were used to compare the differences when appropriate. The area under the receiver operating characteristic curve was calculated, and logistic regression was carried out. The model was constructed by results and regression coefficient of multivariable analysis. All analyses were performed using SPSS (version 26.0) with an alpha level of .05.

## 3 RESULTS

### 3.1 Baseline demographics and clinical characteristics

A total of 229 patients with ICS were enrolled and divided into three groups based on antiplatelet therapy (aspirin group [*n* = 99], clopidogrel group [*n* = 82], and aspirin + clopidogrel group [*n* = 48]). The three groups of ICS patients were generally similar in terms of demographic data, as shown in Table 1.

**TABLE 1:** Demographics and clinical characteristics of the enrolled patients.

Characteristic	Aspirin	Clopidogrel	Aspirin + clopidogrel	*p* value
Sample size, *n*	99	82	48	–
Sex				.091
Male	71 (71.7%)	46 (56.1%)	31 (64.6%)	
Female	28 (28.3%)	36 (43.9%)	17 (35.4%)	
Age (years)	66.97 ± 8.97	67.19 ± 12.68	62.81 ± 10.77	.054
Height, cm	164.02 ± 8.03	162.76 ± 8.11	163.60 ± 7.97	.571
Weight, kg	61.84 ± 10.75	62.29 ± 11.14	65.41 ± 12.01	.176
Education level				.219
Low	24 (24.2%)	25 (30.5%)	18 (37.5%)	
Intermediate	34 (34.3%)	23 (28.0%)	8 (16.7%)	
High	41 (41.5%)	34 (41.5%)	22 (45.8%)	
Medical insurance				.049
Yes	70 (70.7%)	53 (64.6%)	24 (50.0%)	
No	29 (29.3%)	29 (35.4%)	24 (50.0%)	
Marriage				.875
Yes	93 (93.9%)	78 (95.1%)	46 (95.8%)	
No	6 (6.1%)	4 (4.9%)	2 (4.2%)	
Regular exercise				.002
Yes	61 (61.6%)	30 (36.6%)	20 (41.7%)	
No	38 (38.4%)	52 (63.4%)	28 (58.3%)	
Alcohol consumption				.395
Yes	24 (24.2%)	26 (32.1%)	11 (22.9%)	
No	75 (75.8%)	55 (67.9%)	37 (77.1%)	
Tobacco smoking				.873
Yes	24 (26.4%)	21 (27.6%)	11 (23.4%)	
No	67 (73.6%)	55 (72.4%)	36 (76.6%)	

### 3.2 Analysis of adherence in the three treatment groups

**FIGURE 1:**
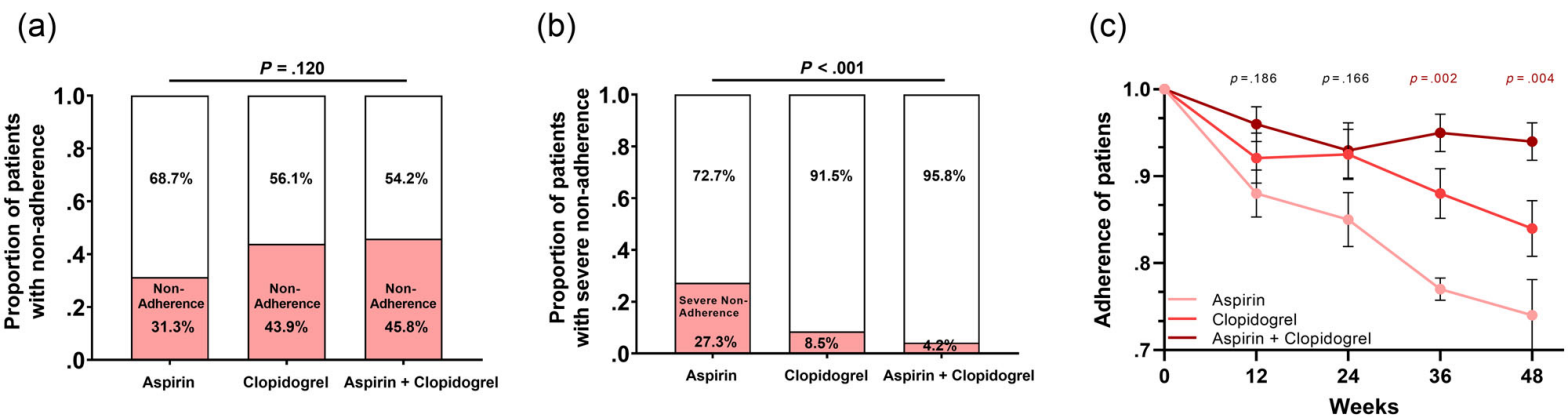
Analysis of adherence in the three treatment groups. (a) There was no significant difference in the proportion of patients with poor adherence (adherence < 100%) among the three groups. (b) A significantly higher proportion of patients in the aspirin group was observed with severe nonadherence (adherence ≤ 75%) than in the other two groups (aspirin vs. clopidogrel vs. aspirin + clopidogrel, 27.3% vs. 8.5% vs. 4.2%, *p* < .001). (c) Adherence in the aspirin group was significantly lower at weeks 36 and 48.

We compared differences in adherence among the three treatment groups, as shown in Figure 1. We found that there was no significant difference in the proportion of patients with poor adherence (adherence < 100%) among the three groups (Figure 1a). However, when we analyzed the proportion of patients with severe nonadherence (adherence ≤ 75%), we found that the proportion of patients in the aspirin group was significantly higher than that in the other two groups (aspirin vs. clopidogrel vs. aspirin + clopidogrel, 27.3% vs. 8.5% vs. 4.2%, *p* < .001; Figure 1b). Therefore, we analyzed the adherence of the three groups over 48 weeks. The results are shown in Figure 1c. Adherence in the aspirin group was significantly lower than that in the other two groups at weeks 36 and 48.

### 3.3 Association between clinical variables and adherence in ICS patients

We analyzed the association between variables and adherence in ICS patients. The results are shown in Table 2. Education level (*p* = .041), medical insurance (*p* < .001), and regular exercise (*p* < .001) habits were related to the adherence of ICS patients.

**TABLE 2:** Differences in patients with adherence and nonadherence.

Characteristic	Adherence	Nonadherence	*p* value
Sample size, *n*	140	89	–
Sex			.667
Male	56 (62.9%)	92 (65.7%)	
Female	33 (37.1%)	48 (34.3%)	
Age (years)	65.47 ± 11.19	67.30 ± 10.37	.216
Height, cm	163.52 ± 7.78	163.42 ± 8.43	.923
Weight, kg	62.72 ± 10.72	62.80 ± 11.96	.957
Education level			.041
Low	36 (25.7%)	35 (39.3%)	
Intermediate	46 (32.9%)	18 (20.2%)	
High	58 (41.4%)	36 (40.4%)	
Medical insurance			<.001
Yes	121 (86.4%)	26 (29.2%)	
No	19 (13.6%)	63 (70.8%)	
Marriage			.838
Yes	133 (95.0%)	84 (94.4%)	
No	7 (5.0%)	5 (5.6%)	
Regular exercise			<.001
Yes	82 (58.6%)	29 (32.6%)	
No	58 (41.4%)	60 (67.4%)	
Alcohol consumption			.234
Yes	34 (24.3%)	28 (31.5%)	
No	106 (75.7%)	61 (68.5%)	
Tobacco smoking			.105
Yes	32 (22.9%)	29 (32.6%)	
No	108 (77.1%)	60 (67.4%)	

### 3.4 Multivariable analysis for adherence and severe nonadherence in ICS patients

**FIGURE 2:**
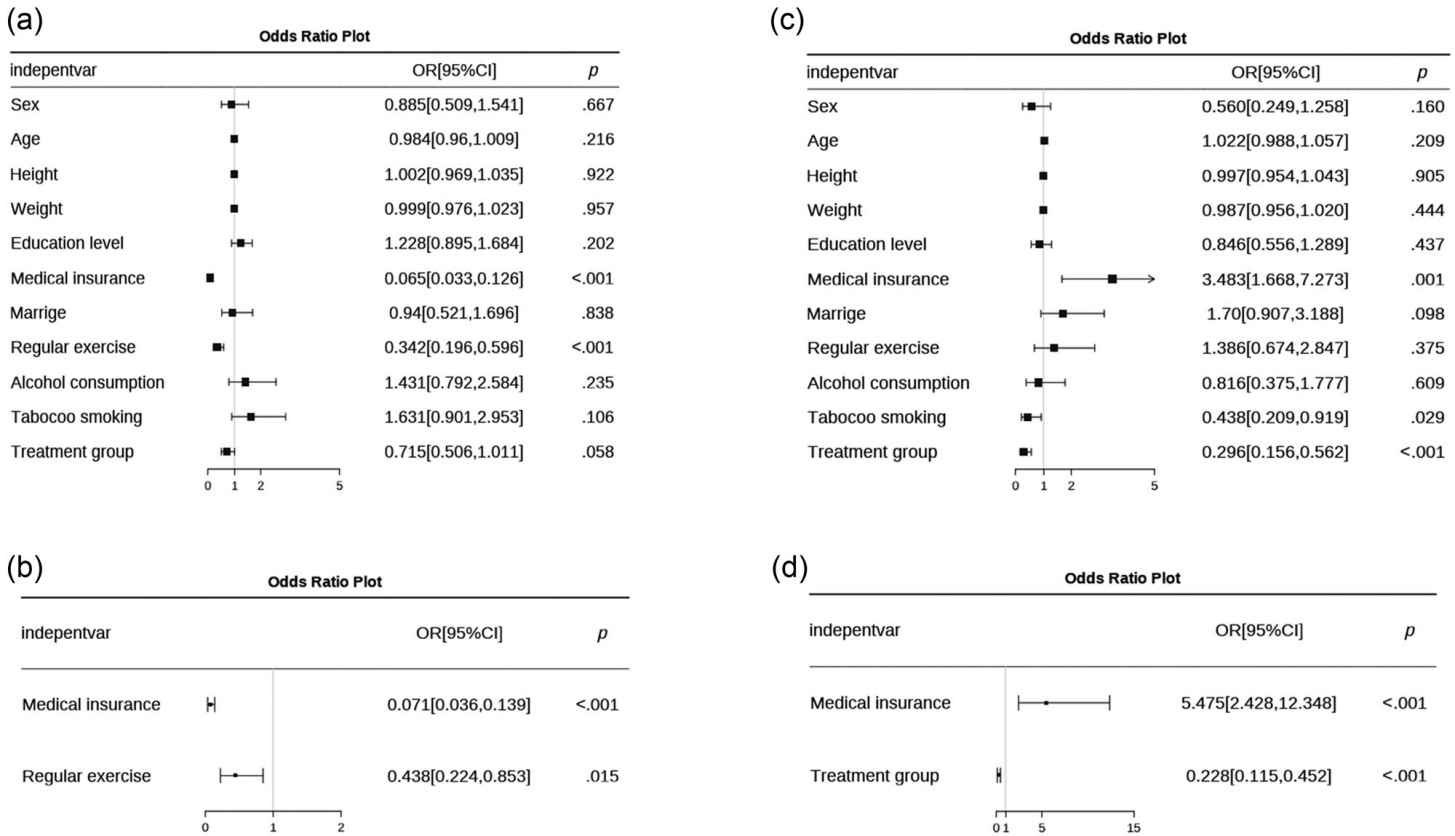
Multivariable analysis for adherence and severe nonadherence in ICS patients. (a) Univariable analyses show that medical insurance (odds ratio [OR] = 0.065, *p* < .001) and regular exercise (OR = 0.342, *p* < .001) are factors associated with adherence in ICS patients. (b) Multivariable analysis indicated that medical insurance (OR = 0.071, *p* < .001) and regular exercise (OR = 0.438, *p* = .015) were independent factors associated with adherence in ICS patients. (c) Univariable analysis revealed that medical insurance (OR = 3.483, *p* = .001), tobacco smoking (OR = 0.438, *p* = .029), and aspirin treatment (OR = 0.296, *p* < .001) were risk factors associated with severe nonadherence in ICS patients. (d) Multivariable analysis indicated that medical insurance (OR = 5.475, *p* < .001) and aspirin treatment (OR = 0.228, *p* < .001) were independent risk factors associated with severe nonadherence.

Univariable and multivariable analyses were conducted to analyze ICS patients with adherence and severe nonadherence, as shown in Figure 2. The results showed that medical insurance (odds ratio [OR] = 0.065, 95% confidence interval [CI]: 0.033–0.126, *p* < .001) and regular exercise (OR = 0.342, 95% CI: 0.196–0.596, *p* < .001) were factors associated with adherence in ICS patients (Figure 2a). Multivariable analysis indicated that both medical insurance (OR = 0.071, 95% CI: 0.036–0.139, *p* < .001) and regular exercise (OR = 0.438, 95% CI: 0.224–0.853, *p* = .015) were independent factors associated with adherence in ICS patients (Figure 2b).

Similarly, we conducted univariable and multivariable analyses, and the results revealed that medical insurance (OR = 3.483, 95% CI: 1.668–7.273, *p* = .001), tobacco smoking (OR = 0.438, 95% CI: 0.209–0.919, *p* = .029), and aspirin treatment (OR = 0.296, 95% CI: 0.156–0.562, *p* < .001) were risk factors associated with severe nonadherence in ICS patients (Figure 2c). However, only medical insurance (OR = 5.475, 95% CI: 2.428–12.348, *p* < .001) and aspirin treatment (OR = 0.228, 95% CI: 0.115–0.452, *p* < .001) were independent risk factors associated with severe nonadherence in ICS patients (Figure 2d).

### 3.5 Subgroup analysis of adherence in the three treatment groups

**FIGURE 3:**
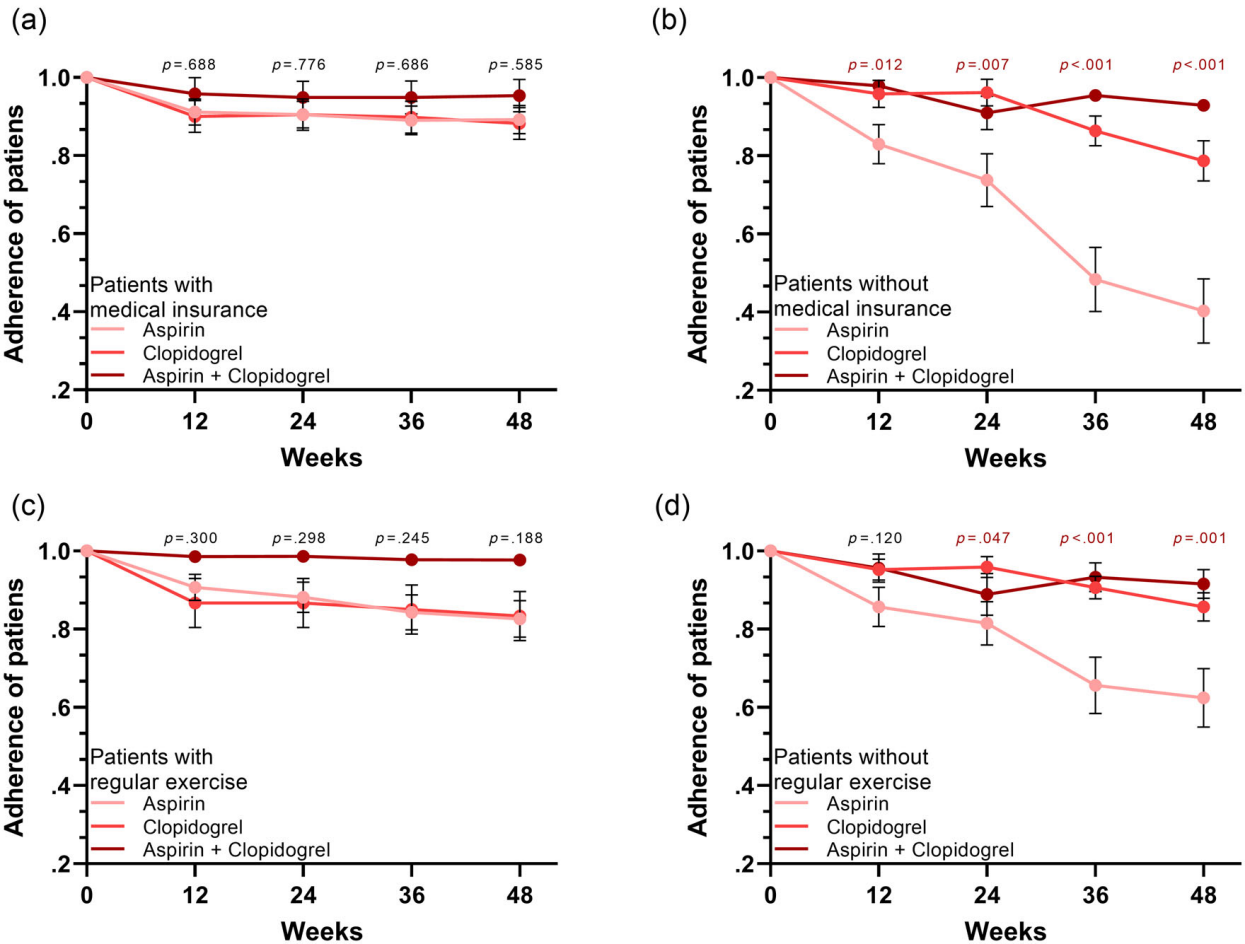
Subgroup analysis of adherence in the three treatment groups. (a) Among patients with medical insurance, no significant differences in adherence were observed between the three treatment groups. (b) Among patients without medical insurance, significantly lower adherence was observed in patients who received aspirin treatment. (c) There was no significant difference in adherence in regular exercise patients in the three treatment groups. (d) For patients without regular exercise, those with aspirin treatment showed the lowest adherence among all patients.

We next investigated the adherence of ICS patients in each subgroup. Of the patients with medical insurance, no significant differences were observed between the three treatment groups (Figure 3a). However, for patients without medical insurance, significantly lower adherence was observed in patients who received aspirin treatment (Figure 3b). Similar trends were observed, with no significant difference in adherence in regular exercise patients in the three treatment groups (Figure 3c). For patients without regular exercise habits, those with aspirin treatment showed the lowest adherence among all patients (Figure 3d).

### 3.6 Construction of a model to predict adherence in ICS patients

**FIGURE 4:**
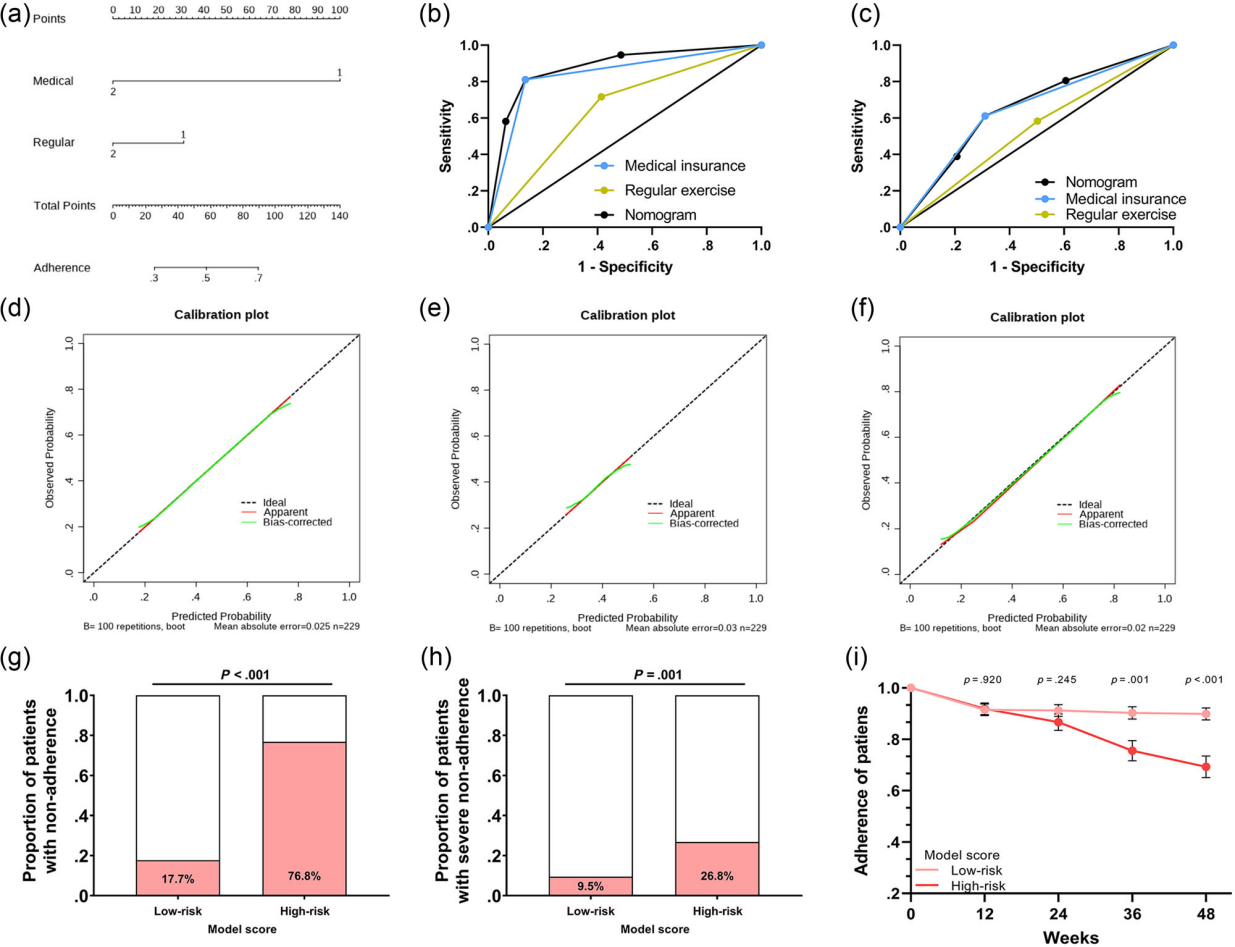
Construction of a model to predict adherence. (a) A nomogram plot was constructed to analyze the relationship between medical insurance and regular exercise on adherence. (b) The areas under the receiver operating characteristic (AUROCs) of medical insurance, regular exercise, and model score for predicting adherence were .786 (95% confidence interval [CI]: .721–.851, *p* < .001), .630 (95% CI: .556–.704, *p* = .001), and .819 (95% CI: .760–.879, *p* < .001), respectively. (c) The AUROCs of medical insurance, regular exercise, and model score for predicting severe nonadherence were .650 (95% CI: .550–.750, *p* = .004), .540 (95% CI: .438–.642, *p* = .442), and .657 (95% CI: .560–.754, *p* = .003), respectively. (d–f) Calibration curves were conducted to validate the effectiveness of medical insurance, regular exercise, and model score‐predicted adherence. (g) A total of 13.3% of patients in the low‐risk group were nonadherent patients, while 53.4% were nonadherent patients in the high‐risk group (*p* < .001). (h) A total of 8.4% of patients in the low‐risk group had severe nonadherence compared with 19.9% in the high‐risk group (*p* = .022). (i) There was a significant difference in the adherence of the two groups of patients within 48 weeks.

We therefore constructed a nomogram to better analyze the relationship between medical insurance and regular exercise on adherence (Figure 4a). Combined with the results of multivariable analysis, the model was constructed as follows: Adherence risk score = 3 × medical insurance (1 if patients had medical insurance, 2 if they did not have medical insurance) + regular exercise (1 if they engaged in regular exercise, 2 if they did not engage in regular exercise). To assess the validity of the adherence risk score model, receiver operating characteristic curves were accessed to evaluate the sensitivity and specificity for predicting nonadherence and severe nonadherence. The results suggest that the adherence risk score is more sensitive and specific for assessing adherence than either medical insurance or regular exercise alone (Figure 4b,c). To further clarify the role of the model in predicting adherence, we constructed calibration curves, as shown in Figure 4d–f. The bias‐corrected adherence risk model can predict the adherence of ICS patients well.

In this regard, we divided patients into low‐risk and high‐risk groups for adherence based on the median adherence risk score. A total of 13.3% of patients in the low‐risk group were nonadherent patients, while 53.4% were nonadherent patients in the high‐risk group (*p* < .001; Figure 4g). Similarly, 8.4% of patients in the low‐risk group had severe nonadherence compared with 19.9% in the high‐risk group (*p* = .022; Figure 4h). Furthermore, there was a significant difference in the adherence of the two groups of patients within 48 weeks, as shown in Figure 4i.

### 3.7 Performance of the model to predict adherence in ICS patients

**FIGURE 5:**
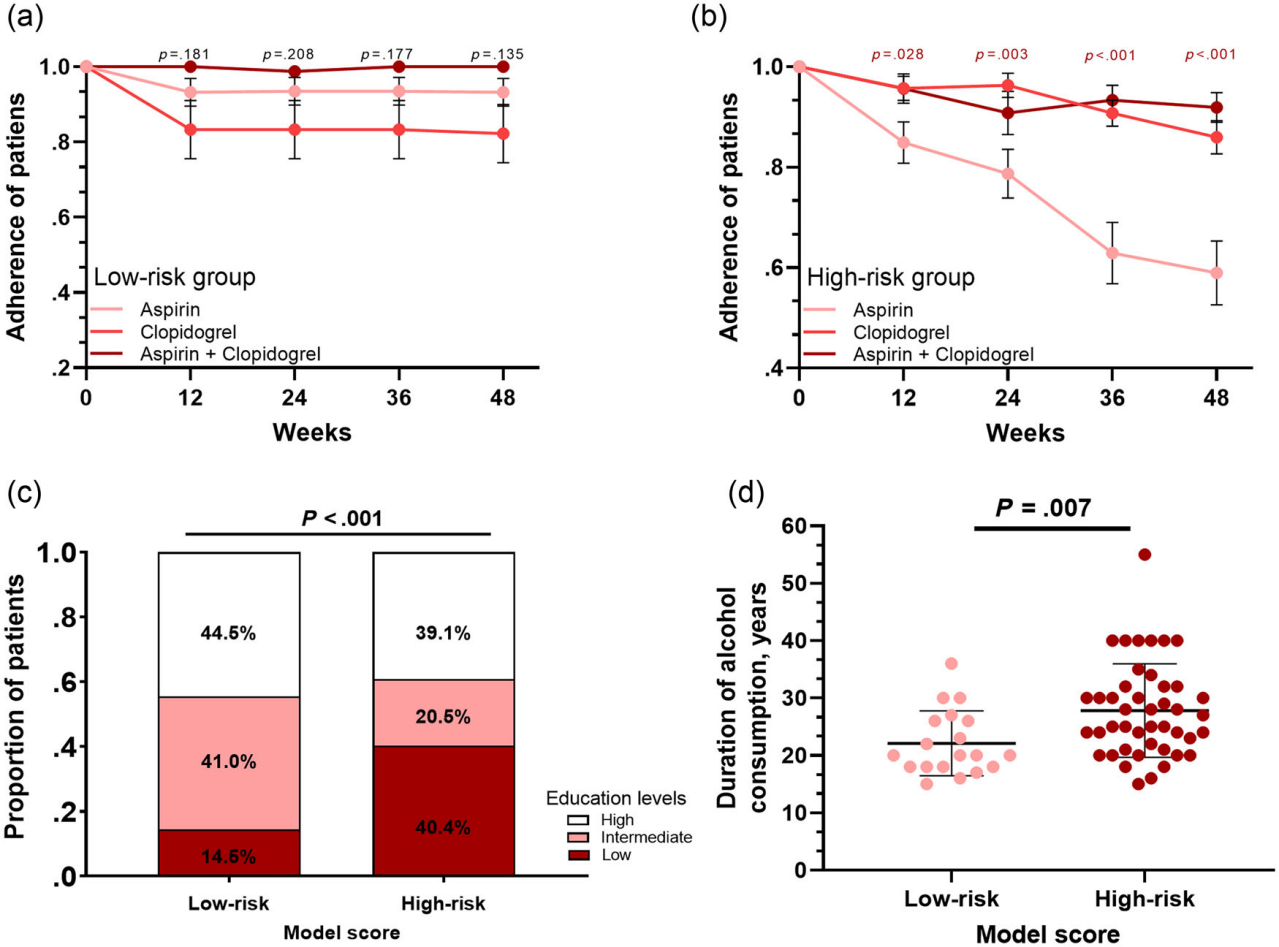
Performance of the model to predict adherence in ischemic cerebral stroke (ICS) patients. (a) No significant difference was observed in the three treatment groups in low‐risk patients. (b) In patients with high risk, the aspirin group showed significantly decreased adherence. (c) Patients at high risk of nonadherence had lower education levels (*p* < .001). (d) The duration of alcohol consumption in low‐risk patients was significantly lower than that in high‐risk patients (*p* = .007).

We next investigated the feasibility of the model score for the adherence of patients in three treatment groups. In patients with low risk, no significant difference was observed in the three treatment groups (Figure 5a). In patients with high risk, aspirin‐treated patients showed significantly decreased adherence compared with the other two groups, as shown in Figure 5b. Interestingly, we also found that the adherence model was significantly correlated with the education level of the patients (*p* < .001; Figure 5c). Patients at high risk of nonadherence had lower education levels than those at low risk of nonadherence. Moreover, among patients with alcohol consumption, the duration of alcohol consumption in low‐risk patients was also significantly lower than that in high‐risk patients (*p* = .007), as shown in Figure 5d.

## 4 DISCUSSION

In this study, we found that there are varying degrees of adherence issues among ICS patients. At the same time, the adherence to different antiplatelet drugs is different. However, only medical insurance and regular exercise were independent factors for adherence. Hence, we constructed an adherence model. By using this model, we divided patients into adherent low‐risk patients and high‐risk patients. For low‐risk ICS patients, there was no difference in adherence to different antiplatelet therapies. However, among high‐risk ICS patients, adherence to aspirin therapy was worse. For those patients, timely adherence education intervention should be given.

Medication adherence is important to ensure the effectiveness of treatment regimens (Brown et al., 2016; Peacock & Krousel‐Wood, 2017). In ICS patients, antithrombotic drugs, including antiplatelet agents and anticoagulants, play an important role in reducing recurrent ischemic stroke (Kamal et al., 2021; Veltkamp et al., 2020). Previous studies have found that patient‐reported poor adherence to medication in patients with atrial fibrillation after ischemic stroke is common and associated with educational levels, absence of heart failure, and smoking history (Tiili et al., 2021). In our study, we confirmed that education level is associated with medication adherence. However, we further found that ICS patients with medical insurance and regular exercise are independent factors associated with better adherence.

Currently, no studies have reported the relationship between medical insurance and adherence in ICS patients. Interestingly, previous studies on osteoporosis indicated that among patients with supplementary health insurance, no difference was observed for drug adherence and extended nonadherence between Black and White patients (Yoo et al., 2013). However, when patients did not have supplementary health insurance, adherence may vary in different populations (Yoo et al., 2013). In our study, we found that patients with health insurance had significantly higher adherence than patients without health insurance. This may involve the medical financial burden problem. Interestingly, our study also found a similar result: there was no significant difference in the adherence of ICS patients among the three treatment regimens when they had health insurance. However, adherence to antiplatelet therapy with aspirin was significantly worse when no health insurance was available in ICS patients. In our study, medical insurance is considered as a factor and a comparison is made between the groups. However, we failed to take gap fee into consideration when comparing medical insurance. The impact of gap fee on patient adherence requires further research to clarify.

The healthy adherer effect is a phenomenon in which patients who adhere to medical therapies tend to pursue health‐seeking behaviors (Andersohn & Willich, 2009; Lee et al., 2018). In our study, we found that regular exercise habits are closely related to good adherence. This may be related to the pursuit of a healthier physical condition in patients with good adherence. A cross‐sectional study with 417 post‐acute myocardial infarction patients who underwent percutaneous coronary intervention indicated that adherence to medication was associated with adherence to lifestyle modification, suggesting the possible presence of the healthy adherer effect in post‐acute myocardial infarction patients (Lee et al., 2018). After further adjusting for health‐related quality of life, the association remained (Lee et al., 2018). Perhaps for ICS patients, early identification of patients with poor adherence and therefore intervention with lifestyle changes and educational programs to improve adherence are important to improve the prognosis of ICS. In addition, our study suggests that healthy adherent effects should be considered in clinical research, especially in studies evaluating the effect of treatment on prognosis. Previous study has also reported that lack of regular exercise is an independent risk factor for poor aspirin adherence in male physicians to prevent myocardial infarction (Glynn et al., 1994). The reason for the association is still unclear. We speculate that maybe the reason here is related to the patient's heavy workload and total income. But more evidence is needed.

In this study, we constructed a risk score model for antiplatelet therapy adherence in ICS patients. Using this model, we can effectively differentiate patients into low‐risk patients with poor adherence and high‐risk patients. For high‐risk patients, there were significant differences in the adherence to different antiplatelet drugs. Interestingly, the model was also found to be strongly associated with education level and the duration of alcohol consumption. For high‐risk patients, it is important to identify their poor adherence behavior early and provide adherence education intervention.

Our study has some limitations. First, this is a single‐center study. Second, the relatively small sample size may bring some bias. Our study was limited to patients in Guangxi, China. The level of adherence to antiplatelet therapy in patients living at different developmental levels needs to be further evaluated. Moreover, the number of missing tablets was acquired by patient self‐report, which may introduce some bias in the results. Our study could also benefit from extra data from different sites and differences in ethnicities. The effect of adherence interventions on high‐risk patients based on the adherence model needs further research.

## 5 CONCLUSION

The adherence to different antiplatelet drugs is different. However, only medical insurance and regular exercise were independent factors for antiplatelet therapy adherence. We therefore constructed a model, and the model score could effectively risk‐stratify patients with poor adherence. For patients with high model scores, timely intervention is necessary.

## AUTHOR CONTRIBUTIONS

Jie Zhong and Yueqiang Hu conceptualized and designed the study. Limei Diao and Wei Chen curated the data. Qianchao He and Deqing Huang analyzed the data. Jie Zhong and Yuguang Gao drafted the manuscript. All authors reviewed and approved the final manuscript.

## CONFLICT OF INTEREST STATEMENT

The authors declare no conflicts of interest.

### PEER REVIEW

The peer review history for this article is available at https://publons.com/publon/10.1002/brb3.2982.

## Data Availability

All relevant data are included in the article and materials are available on reasonable request from the authors.
